# “Yes, but will it work for *my* patients?” Driving clinically relevant research with benchmark datasets

**DOI:** 10.1038/s41746-020-0295-6

**Published:** 2020-06-19

**Authors:** Trishan Panch, Tom J. Pollard, Heather Mattie, Emily Lindemer, Pearse A. Keane, Leo Anthony Celi

**Affiliations:** 1 Division of Health Policy and Management, Harvard T.H. Chan School of Public Health, Boston, MA USA; 2 Wellframe Inc., Boston, MA USA; 3 Laboratory for Computational Physiology, Massachusetts Institute of Technology, Cambridge, MA USA; 4 Department of Biostatistics, Harvard T.H. Chan School of Public Health, Boston, MA USA; 5 NIHR Biomedical Research Centre at Moorfields Eye Hospital and UCL Institute of Ophthalmology, London, UK; 6 Division of Pulmonary, Critical Care and Sleep Medicine, Beth Israel Deaconess Medical Center, Boston, MA USA

**Keywords:** Health policy, Databases

## Abstract

Benchmark datasets have a powerful normative influence: by determining how the real world is represented in data, they define which problems will first be solved by algorithms built using the datasets and, by extension, who these algorithms will work *for*. It is desirable for these datasets to serve four functions: (1) enabling the creation of clinically relevant algorithms; (2) facilitating like-for-like comparison of algorithmic performance; (3) ensuring reproducibility of algorithms; (4) asserting a normative influence on the clinical domains and diversity of patients that will potentially benefit from technological advances. Without benchmark datasets that satisfy these functions, it is impossible to address two perennial concerns of clinicians experienced in computational research: “the data scientists just go where the data is rather than where the needs are,” and, “yes, but will this work for my patients?” If algorithms are to be developed and applied for the care of patients, then it is prudent for the research community to create benchmark datasets proactively, across specialties. As yet, best practice in this area has not been defined. Broadly speaking, efforts will include design of the dataset; compliance and contracting issues relating to the sharing of sensitive data; enabling access and reuse; and planning for translation of algorithms to the clinical environment. If a deliberate and systematic approach is not followed, not only will the considerable benefits of clinical algorithms fail to be realized, but the potential harms may be regressively incurred across existing gradients of social inequity.

## Introduction

In 2012 Krizhevsky et al. presented an image recognition algorithm at the Neural Information Processing Systems conference that delivered performance “considerably better than the previous state-of-the-art results” on the ImageNet dataset—a collection of over 15 million images belonging to roughly 22,000 categories^1,2^. “AlexNet” is considered by many to be a landmark in machine learning, helping to drive the recent surge of interest in deep learning^3^. Typically, algorithms such as AlexNet are developed upon curated data, which also serves as a common standard for evaluation—a benchmark dataset. Such benchmark datasets have a powerful normative influence: by determining how the real world is represented in data, they define which problems will first be solved by algorithms built using the datasets and, by extension, who these algorithms will work *for*. Whilst much of the credit for such landmarks in machine learning has accrued to the creators of algorithms, it is important that the contributions of the creators of the datasets that enable these formative advances are also recognized^4^.

Recently, ImageNet Roulette, an application that tested the performance of an image classifier built on ImageNet^5^ revealed that “while the program identified white individuals largely in terms of occupation or other functional descriptors, it often classified those with darker skin solely by race,” which prompted recalibration of ImageNet itself^6,7^. The ImageNet dataset was built using human annotated images collated from across the internet. Biases of the human annotators were encoded into the dataset and, as a result, unwittingly within the algorithms that were built upon it. ImageNet, like all existing benchmark datasets, was developed opportunistically at a time when the principal aim was seeding development in machine learning rather than longer term practical considerations such as fairness^8^.

ImageNet, both its successes and failures, provides lessons for data intensive research. Within healthcare, there is a need for clinical and research communities to take a more active role in the development and oversight of benchmark datasets. Given the Covid-19 pandemic and increasing calls for open datasets to enable the creation of machine learning models, there is particular urgency to define best practice in this area^9^. It is desirable for these datasets to serve a number of functions, including: (1) enabling the creation of clinically relevant algorithms; (2) facilitating like-for-like comparison of algorithmic performance; (3) ensuring reproducibility of algorithms^10^; (4) asserting a normative influence on the clinical domains and diversity of patients that will potentially benefit from technological advances (see also Box 1). The latter function is necessarily subjective: it is for national and local health systems to determine which priorities are relevant to the populations they serve. Without benchmark datasets that satisfy these functions, it is impossible to address two perennial concerns of clinicians experienced in computational research: “the data scientists just go where the data is rather than where the needs are,” and, “yes, but will this work for my patients?”

If algorithms are to be developed and applied for the care of patients, then it is prudent for the research community to create benchmark datasets proactively, across specialties. As yet, best practice in this area has not been defined, but the task necessarily involves the synthesis of engineering, legal, clinical, and health systems expertize. Broadly speaking, efforts will include design of the dataset; compliance and contracting issues relating to the sharing of sensitive data; enabling access and reuse; and planning for translation of algorithms to the clinical environment. While there are no one-size-fits-all solutions in any of these areas, there are common topics that we would expect to feature prominently when developing best practice.

Box 1 Desirable functions of a benchmark dataset. 1. Enabling the creation of algorithms to perform a desired task.

2. Facilitating like-for-like comparison of algorithmic performance.

3. Ensuring reproducibility of algorithms.

4. Asserting a normative influence on the clinical domains and diversity of patients that will potentially benefit from algorithms.

## Design

The content of benchmark datasets determines which clinical questions might be answered using the data, which patients and diseases are represented within the data, and in turn which groups might benefit from algorithms developed upon it. To ensure that clinically relevant priorities are at the forefront, design ideally involves clinicians and health policy specialists (so that national and regional health system priorities are represented). Though it may be argued that such an approach slows progress and increases the costs of algorithm development, these costs are offset by the downstream benefits of improved relevance to health systems and likelihood of adoption in clinical practice. It is important to be mindful that in rare diseases (where, by definition, there is a scarcity of data) or for diseases affecting marginalized populations (such as those with substance misuse), achieving representation in benchmark datasets may be challenging, even though these cases will likely be represented in health priorities.

Parallel to the selection of content, structural design requires careful thought. While it may be beneficial to structure a dataset for optimal ease-of-use, there may also be value in releasing data in its native form to allow algorithms to be more easily translated back to the clinical environment. The ability to reuse publicly developed code within local environments can be a motivation for data custodians to share, as was the case for the eICU Collaborative Research Database^11^. When creating multicenter datasets, common data models that allow structure and terminology to be linked across data sources are also a consideration. There is often a desire to intensively “clean” data before sharing. Doing so can introduce unwanted biases, so in general we believe that steps such as imputation of missing data should be avoided, or at least treated with caution.

Given the complexity of creating the ideal benchmark dataset, the release of multiple, sequentially improved versions of a benchmark dataset is advisable. As the user community generates knowledge using the data that pushes the frontier of medicine forward, their feedback should galvanize the dataset creators to make improvements. Algorithms may be found to suffer from common areas of failure related to systematic differences between the data in benchmark datasets and the real world which adversely affects performance of algorithms in real-world applications—for example degradation of performance in specific racial groups as previously mentioned or issues with image capture in radiology that limit generalizability. In these cases benchmark datasets should be actively designed to address the areas of failure. For example, in the aforementioned applications: greater diversity of racial representation or representation of modalities of image capture reflective of clinical practice^12,13^.

## Compliance and contracting

In many healthcare institutions, policies, and infrastructure to support data sharing will need development. Clinician champions should work with information security and corporate leadership to create a framework that supports the creation of benchmark datasets. In almost all cases, de-identifying data will be a requirement. Deidentification, at the very least, typically involves removing elements such as patient names, ID numbers, contact details, and exclusion of rare cases^14,15^. In the United States, identifiable patient data is covered by the Health Insurance Portability and Accountability Act (HIPAA) Privacy Rule, which outlines 18 identifiers that constitute protected health information (PHI). If PHI is removed through deidentification, the Privacy Rule does not restrict the use or disclosure of health information.

Ethics committee approval or exemption should be sought for creating the benchmark dataset, a task that may be simplified given deidentification. Consent from individual patients may be impractical or impossible to obtain with retrospective data, but patient level consent is the ideal. “Opt-in” models, though lighter touch, may result in only the most proactive patients engaging. The inherent risk is that algorithms will only work well in these proactive populations, compounding inequities in regard to age, ethnicity, and biological sex^16^. In 2016 the UK’s National Data Guardian concluded that “opt-out” models would be the most appropriate for collection and secondary use of National Health Service data^17^ and from 2018, a national data opt-out was instituted. For opt-out approaches to be successful, healthcare systems using them must implement processes for ease-of-use, transparency, governance, and accountability. Central to these processes will be the need to demonstrate the public and social benefits of any potential data use before permission is given. Furthermore, from a software engineering point of view, operationalizing consent by effectively marking data with consent metadata updated in real time should be a research and development priority.

## Access and reuse

The FAIR (findable, accessible, interoperable, and reusable) principles for good practice in data management and stewardship should be applied for all benchmark datasets^18^. Access may need to be limited to approved users, but it is important to note that this concept is distinct from enabling discovery and formal citation. Cloud hosting can drastically reduce the technical challenges for healthcare organizations in making benchmark datasets available to an international research community. Beyond hosting the data, however, there are additional challenges in creating an ecosystem of collaborative investigation around the dataset.

Our experience in sharing datasets such as MIMIC-III, a critical care database that is widely used for machine learning research, has emphasized the importance of providing a direct gateway of communication between the research community and those who are involved in the data generation process (for example, nurses who chart data and teams responsible for disease coding)^19^. Ensuring that documentation is continuously updated and handling questions and answers publicly, rather than in private channels such as emails, help to make the demands of user support more manageable. Efforts should be taken to create interdisciplinary relationships between clinical experts and computational and statistical scientists. The use of hackathons and datathons can be effective in creating these relationships^20,21^, and open source code can facilitate analysis and support fully reproducible studies^22^.

## Translation

Whilst there are recommendations on best practice for translating algorithmic potential into clinical impact^23^, few organizations have meaningfully implemented machine learning algorithms in daily practice. The reality is that most healthcare organizations do not have the expertize or resources to develop machine learning algorithms beyond proof of concept themselves and as such are beginning to rely on third party partners including prominent technology companies who have entered this area^24^.

Algorithms trained on private data or public benchmark datasets will need to be validated *locally* to ensure that promised algorithmic performance is delivered for local patients. In fact, in recent guidance, the Radiological Society of North America^25^ stipulated that an external test set should be used for final statistical reporting of algorithms in research. To implement this guidance in research or to validate algorithmic performance for translation of research into practice there is a common need: local benchmark datasets. This necessitates a process of creation and curation of local benchmark datasets for individual healthcare organizations, or more realistically collections thereof, that is analogous to the creation of national or global benchmark datasets as previously described.

Whilst it appears desirable for all benchmark datasets to be highly curated to improve the efficiency of creating models, the reality is that once these models are created and validated, they will have to be applied to real world health data which is typically less clean and less complete^26^. As such the performance of algorithms on benchmark datasets will typically not reflect real world performance. For this reason, *local* benchmark datasets should reflect operational data such that live, unprocessed data can be run through algorithms to validate performance at the frontlines.

Even so, these measures are not a guarantee of *enduring* performance. The performance of an algorithm will potentially decrease over time: for example, an algorithm that predicts acute kidney injury was implemented at several Veterans Administration hospitals. Within a few years, the model started overestimating risk, and the magnitude of overestimations increased over time^27^. Similarly, Google flu trends initially set a performance benchmark in 2010, but by 2013, shifts in the manner that the public searched for terms related to flu on Google search eroded the performance of the algorithm^28^. This need for updating in the face of changes in the data generating process is a common need for all algorithms.

## Conclusion

Benchmark datasets are essential for computational research in healthcare. These datasets should be created by intentional design that is mindful of social and health system priorities. If a deliberate and systematic approach is not followed, not only will the considerable benefits of clinical algorithms fail to be realized, but the potential harms may be regressively incurred across existing gradients of social inequity^29^.
